# Response to Hydralazine-Valproate in a Patient with Mycosis Fungoides

**DOI:** 10.1155/2010/657579

**Published:** 2010-03-21

**Authors:** Alfonso Dueñas-Gonzalez, Maria Teresa Vega, Déborah Martinez-Baños, Linda García-Hidalgo, Pedro Sobrevilla

**Affiliations:** ^1^Unidad de Investigación Biomédica en Cancer, Instituto de Investigaciones Biomédicas, Instituto Nacional de Cancerología, UNAM, San Fernando 22, Tlalpan 14080, Mexico City, Mexico; ^2^Department of Dermatology, Instituto Nacional de Cancerología, Tlalpan 14080, Mexico City, Mexico; ^3^Department of Hematology, Instituto Nacional de Ciencias Médicas y Nutrición Salvador Zubirán, Tlalpan 14080, Mexico City, Mexico; ^4^Department of Dermatology, Instituto Nacional de Ciencias Médicas y Nutrición Salvador Zubirán, Tlalpan 14080, Mexico City, Mexico; ^5^Department of Hematology, Instituto Nacional de Cancerología, Tlalpan 14080, Mexico City, Mexico

## Abstract

Histone deacetylase (HDAC) inhibitors have shown significant activity in the treatment of cutaneous T-cell lymphomas (CTCL). The epigenetic alterations of CTCL not only are limited to altered histone acetylation but also include aberrant DNA gene methylation hence, the combination of an HDAC inhibitor with a DNA demethylating agent is a promising therapy to be tested. Here we report a mycosis fungoides patient having a dramatic response to hydralazine and valproate, two repositioned drugs as HDAC and DNA methylation inhibitors, respectively.

## 1. Introduction

Mycosis fungoides (MF) is a rare disease that represents the most common type of primary CTCL, which is characterized by a clonal proliferation of malignant lymphocytes in the skin. Typically, the natural history of MF is indolent. Symptoms of the disease may present for long periods, an average of 2 to 10 years, as waxing and waning cutaneous eruptions prior to biopsy confirmation [1]. Currently, skin-directed treatment regimens, like phototherapy and corticosteroids, are commonly used in early stages; while for advanced diseases systemic treatments include bexarotene, denileukin diftitox, lenalidomide, toll-like receptor agonists, pralatrexate, bortezomib, as well as cytotoxic chemotherapy. Curative modalities, however, have thus far proven elusive, with the possible exception of patients with minimal disease confined to the skin [2]. Epigenetic therapy based on DNA methyltransferase (DNMT) and HDAC inhibitors is at present widely evaluated in a number of malignant diseases [3]. CTCL has been found to overexpress several HDACs which include HDAC1, HDAC2, and HDAC6 [4] and several clinical trials have examined the sensitivity of CTCL to HDAC inhibitors. Vorinostat, panobinostat, belinostat, and romidepsin have all shown efficacy in indolent and advanced stages of CTCL in patients who have failed prior systemic therapies. Vorinostat yields response rates of 25%–30% in advanced stages (MF stage IIB–IVB) [5]. Belinostat has shown efficacy in a phase II clinical trial against recurrent and refractory CTCL with PRs in two of eight patients with MF [6]. Romidepsin has resulted in an overall response rate of 31%, with PRs reported in plaque/patch and tumor stages of MF [7]. Panobinostat has also demonstrated a response rate in six out of 10 patients with advanced-stage CTCL, with two CRs and four PRs [8]. Although objective responses, that is, complete or partial, are not reached by all CTCL patients, many still benefit from treatment by achieving stable disease and/or pruritus relief [5–8]. On the other hand, malignant T cells of patients with CTCL also display widespread promoter hypermethylation associated with inactivation of several tumor suppressor genes involved in DNA repair, cell cycle, and apoptosis signaling pathways; however, no clinical studies have been reported with DNMT inhibitors in this neoplasia [9]. 

Hydralazine and magnesium valproate (HV) are DNMT and HDAC inhibitors, respectively. Clinically, these drugs decrease global DNA methylation and HDAC activity in the peripheral blood of patients, as well as upregulation of genes in primary breast cancer tumors [10]. Likewise, in a pilot study these epigenetic drugs were able to overcome chemotherapy resistance in refractory solid tumor patients regardless of tumor type and chemotherapy schedule [11]. Overall, this information may suggest that HV could be an effective therapy for CTCL.

## 2. Case Report

**Figure 1:**
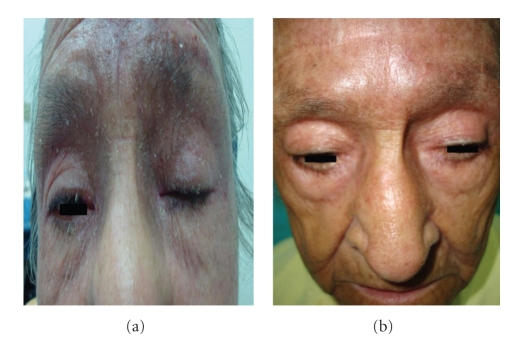
The status of the disease before starting HV is shown in Figure 1(a), and after treatment in Figure 1(b).

This 74-year-old female patient was seen in September 2007 with a three-month history of disseminated itching cutaneous erythematous hyperpigmented plaques affecting the face, chest, and arms to then disseminate to most of body surface except in palms, soles, and scalp. A skin biopsy was reported as reticular erythematous mucinosis and treatment was started with topical steroids and hydroxychloroquine with no response. In December 2007 the treatment was changed to thalidomide, again with no response. In April 2008 a new skin biopsy was reported as compatible with Mycosis Fungoides by having an atypical CD4 lymphocytic infiltrate. Extension studies which included cervical, thoracic, abdominal, and pelvic CT scan as well as a bone marrow biopsy were negative so she was staged as IIIA. Right after, she started treatment with oral methotrexate at increasing doses starting at 10 mg/m^2^ weekly up to 40 mg/m^2^ until September 2008 when she achieved a maximum response in the size and extension of plaques. However, no improvement in itching was achieved. In October 2008 her lesions worsened affecting almost 80% of body surface plus the presence of ulcerated and nonulcerated nodules in the scalp. By December 2008, she entered into a clinical protocol with single agent enzastaurine. Nonetheless, after the first course of treatment she had progression with lesions affecting almost the totality of body surface area. In January 2009 after an extensive information and discussion with the patient and her daughter, she accepted to receive hydralazine and magnesium valproate in a compassive manner. Representative pictures of the disease status at this point and after treatment are shown in Figures 1(a) and 1(b). 

The epigenetic therapy consisted on magnesium valproate at 30 mg/Kg and hydralazine at a total daily dose of 182 mg as she was typed as rapid acetylator (slow-acetylators receive a dose of 83 mg/day, the acetylator phenotype is performed with the sulfametazine test, where patients take P.O. sulfametazine and urine is collected by 6 hours, then the ratio of acetylated/nonacetylated metabolites is measured). These medications are formulated in slow-release tablets. Within the next week she started to feel decreasing in the severity of pruritus and decreasing in size and erythema of plaques. After two weeks the patient reported complete resolution of pruritus and there was no need of taking gabapentin due to the disappearance of neuropathic symptoms in both hands. In the ensuing weeks the erythrodermia vanished only remaining a slightly hyperpigmented skin in the previous diseased areas. This dramatic response was however accompanied by side effects. Soon after starting medication the patient complained of somnolence (which did not interfere with the normal-life activities), fatigue, nausea, dysgeusia, anorexia, and weight loss. In addition, the patient had dehydration and a paradoxical hypertension which required antihypertensive drugs. Most likely, both factors conditioned acute renal failure (a maximum creatinine of 6.5 mg/100 mL which required hospitalization for hydration and pharmacological control of hypertension.) Ten days after, serum creatinine returned to normal. This episode led to withdrew hydralazine and valproate. In March 2009 the patient continued taking no epigenetic drugs and commenced again with pruritus and mild erythrodermia in previously affected areas, hence valproate only was restarted and after three weeks the patient became again asymptomatic. Currently, she remains free of symptoms and the skin condition with no signs of active disease at 14 months of treatment, except for a persistent but decreasing dandruff.

## 3. Discussion

The dramatic clinical response to the combination of hydralazine and valproate is in line with the activity against CTCL of HDAC inhibitors as drug class [5–8]. This result is not unexpected as valproic acid has shown yet to be less potent than other HDAC inhibitors to have remarkable antitumor activity either alone or associated with chemotherapy against a number of neoplasms, particularly hematological ones [12–15]. On the other hand, hydralazine—a weak nonnucleoside DNA methylation inhibitor [16] has shown synergy with valproic acid in regard to gene reactivation and antitumor effects [17]. Though the activity of this combination is limited to the present case, the response rate seemed to occur faster than that achieved with vorinostat which is reported to range from 3.6 to 21.9 weeks [5], this may suggest that adding a DNA methylation inhibitor could be advantageous by facilitating the reactivation of tumor suppressor genes silenced by epigenetic mechanisms [9]. This concept has been widely proven in experimental systems were both DNMTs and HDACs participate in the epigenetic silencing of genes [18]. Nevertheless, this combination is not devoid of side effects. In previous studies with hydralazine and valproate added to chemotherapy or chemoradiation, the most frequent and significant toxicity is hematological followed by somnolence which usually is transient, as well as fatigue and anorexia [10, 11]. Unexpectedly, the patient presented paradoxical hypertension which in our knowledge has not been reported with this agent or in combination with valproate which was coincidental with dehydration causing transient renal failure. Nevertheless, because of the rapid response to the treatment, a tumor lysis syndrome cannot be ruled out as the cause of the transient renal failure. In conclusion, this case report is illustrative on the activity of known drugs which have been repositioned in cancer therapeutics [19, 20]. Currently, a phase II trial with this combination is ongoing in CTCL. If the activity and safety of this therapy is proven, it will definitively be a great contribution for the management of this neoplasia worldwide because at difference of the other HDAC inhibitors which have been developed following the current approach of DDD (Drug Design & Development) and have a prohibitive cost for many cancer patients, repositioned old drugs as a rule will have lower price.
